# Initial Calcium Derangements in Major Trauma and Outcomes

**DOI:** 10.1001/jamanetworkopen.2026.0083

**Published:** 2026-02-25

**Authors:** Steven G. Schauer, Susannah E. Nicholson, Julie A. Rizzo, Franklin L. Wright, Allyson A. Arana, Michael D. April, Lauran Barry, James Bynum, Alex C. Cheng, Andrew D. Fisher, Jennifer M. Gurney, R. Jimena Huaman, Donald H. Jenkins, Brian J. Kirkwood, Bethany C. Lambert, Brit J. Long, Fabiola Mancha, Melody A. Martinez, Michael A. Meledeo, Jessica Mendez, Isabel Newton, Stacy A. Shackelford, Dayana Sifuentes, Vikhyat S. Bebarta, Andrew P. Cap

**Affiliations:** 1 US Army Institute of Surgical Research, JBSA Ft Sam Houston, Ft Sam Houston, Texas; 2 Department of Emergency Medicine, Brooke Army Medical Center, JBSA Ft Sam Houston, Ft Sam Houston, Texas; 3 Center for Combat and Battlefield (COMBAT) Research, University of Colorado School of Medicine, Aurora; 4 Department of Surgery, University of Texas Health at San Antonio, San Antonio; 5 Department of Surgery, Brooke Army Medical Center, JBSA Ft Sam Houston, Ft Sam Houston, Texas; 6 Department of Surgery, University of Colorado School of Medicine, Aurora; 7 59th Medical Wing, JBSA Lackland, Lackland, Texas; 8 Department of Biomedical Informatics, Vanderbilt University Medical Center, Nashville, Tennessee; 9 Department of Surgery, University of Texas Health Science Center at Houston, Houston; 10 Joint Trauma System, Defense Health Agency, JBSA Fort Sam Houston, Texas; 11 The Metis Foundation, San Antonio, Texas; 12 University of Virginia Medical Center, Charlottesville; 13 Department of Surgery, United States Air Force Academy, Colorado Springs, Colorado; 14 Department of Emergency Medicine, University of Colorado School of Medicine, Aurora; 15 Department of Medicine, Uniformed Services University, Bethesda, Maryland

## Abstract

**Importance:**

Calcium derangements frequently occur in the setting of transfusions, but limited data suggest that derangements may occur from the trauma itself. Existing data suggest worse outcomes for trauma patients who present with calcium derangements.

**Objective:**

To determine the incidence of calcium derangements upon emergency department (ED) arrival after major trauma and associated outcomes.

**Design, Setting, and Participants:**

This prospective, multicenter cohort study was conducted from 2022 to 2024 at 3 American College of Surgeons–verified level I trauma centers. All of the centers are regional receiving centers in large cities. Participants who arrived within 24 hours of injury and met the institutional criteria for the highest level of trauma activation were enrolled. Ionized calcium measurements were obtained immediately upon arrival to the ED. A normal reference range of 4.4 mg/dL to 5.2 mg/dL (to convert to millimoles per liter, multiply by 0.25) was used to define hypocalcemia, eucalcemia, and hypercalcemia.

**Exposure:**

Major trauma and evaluation at a verified trauma center.

**Main Outcomes and Measures:**

The primary outcome was the incidence of calcium derangements upon ED arrival. Descriptive and inferential statistics were used to compare the cohorts.

**Results:**

A total of 1270 participants (median [IQR] age, 35 [25-52] years; 999 male [79%]) were enrolled with an overall. Motor vehicle collisions followed by firearms were the most common injury mechanisms. There were 282 patients (22%) who were hypocalcemic, 925 patients (73%) who were eucalcemic, and 57 (5%) who were hypercalcemic. Mortality at 24 hours was 11.9% (34 patients) for hypocalcemia, 4.3% (40 patients) for eucalcemia, and 22.8% (13 patients) for hypercalemia. Mortality increased as the derangements became more extreme, in a U-shaped relationship. The median (IQR) injury severity score was 21 (10-29) for hypocalcemia, 14 (5-25) for eucalcemia, and 22 (13-29) for hypercalcemia. Administration of any blood product during the first 24 hours was 64.1% (184 patients) for hypocalcemia, 31.5% (291 patients) for eucalcemia, and 66.7% (38 patients) for hypercalcemia.

**Conclusions and Relevance:**

In this cohort study of trauma patients, hypercalcemia was less common than hypocalcemia; however, hypercalcemia was associated with worse mortality at all time points. Blood product consumption was higher among those with hypercalcemia or hypocalcemia compared with eucalcemia but was similar among those with any calcium derangement. Prospective interventional trials are needed to understand the implications of empiric treatment.

## Key Points

**Question:**

What is the incidence of calcium derangements upon emergency department arrival after major trauma, and are they associated with outcomes?

**Findings:**

This cohort study found that among 1270 participants arriving to 3 level I trauma centers, 22% were hypocalcemic and 5% were hypercalcemic. Mortality at 24 hours was 11.9% for hypocalcemia, 4.3% for eucalcemia, and 22.8% for hypercalcemia.

**Meaning:**

These findings suggest that hypercalcemia was less common than hypocalcemia but was associated with worse mortality; additional research is needed to determine the underlying pathophysiology.

## Teaser

This cohort study examines the incidence of calcium derangements upon emergency department arrival after major trauma and associated outcomes.

## Introduction

Hypocalcemia occurs frequently in critically ill patients, including severely injured trauma patients.^1,2^ Hypocalcemia is associated with massive resuscitation with blood products and chelation of serum calcium by the citrate in anticoagulant storage solutions.^3^ Data from the military suggest that the trauma in and of itself may be associated with hypocalcemia prior to administration of blood products. Conner et al^3^ prospectively collected data on casualties arriving to one forward surgical facility in Afghanistan. In their study, they assessed 101 patients, of whom 55 (54.5%) were found to be hypocalcemic on arrival to the forward surgical facility.^3^ Nguyen et al^4^ analyzed data from the Department of Defense Trauma Registry and found that 26% of the presenting casualties who had an ionized calcium (iCa) measured in the emergency department (ED) had a calcium derangement. In addition, Nguyen et al^4^ found associations between calcium derangements and elevated international normalized ratio, acidosis, tachycardia, hypotension, depressed Glasgow Coma Scale score, and receipt of blood products, suggesting important physiological interactions with calcium derangements. A 2024 systematic review and meta-analysis analyzed data from 9 studies reporting iCa upon arrival to the ED.^5^ The authors analyzed 14 studies with a total of 3711 patients and found that hypocalcemia upon arrival was common, with a mean arrival value of 1.08 mmol/L. However, most of those studies were retrospective in design. Moreover, that systematic review did not report on the incidence of hypercalcemia, which may also be a clinically important finding.^6^

There exist limited data to guide empiric administration of calcium in the setting of major hemorrhage, particularly when hypocalcemia from the injury itself may exist prior to receiving blood products. The current Joint Trauma System Clinical Practice Guideline on Damage Control Resuscitation^7^ recommends that calcium be empirically administered in patients with hemorrhagic shock during or immediately after transfusion of the first use of a blood product and with every 4 units of product thereafter. Similar recommendations are noted in the Damage Control Resuscitation in Prolonged Field Care^8^ and the Advanced Trauma Life Support guidelines.^9^ However, none of these guidelines accounts for potential calcium derangements that may be present prior to the transfusion of blood products. More high-quality data are needed to understand calcium derangements upon arrival to the ED. We determined the incidence of calcium derangements upon arrival after major trauma and associated outcomes.

## Methods

### Ethics

During the conduct of this cohort study, Brooke Army Medical Center (BAMC) and University of Colorado Hospital (UCH) operated under US Army Institute of Surgical Research protocol H-21-034, which the US Army Medical Research and Development Command institutional review board approved. University Hospital (UH) operated under protocol 20220226EX, which was approved by the University of Texas Health at San Antonio institutional review board. Both protocols underwent second-level review by the Defense Health Agency Office of Human Research Oversight. Both sites requested, and obtained, Health Insurance Portability and Accountability Act waivers for accessing patient data. Given the observational, minimal risk nature of our study, and the impracticability of obtaining written consent, we requested and were granted a waiver of informed consent. We adhered to the Strengthening the Reporting of Observational Studies in Epidemiology (STROBE) reporting guidelines for the analysis and reporting of this study.

### Setting

The study took place at 3 level I trauma centers—BAMC, UH at the University of Texas Health at San Antonio, and the UCH at the University of Colorado School of Medicine from 2022 to 2024. The study was led from the US Army Institute of Surgical Research, which is physically adjacent to BAMC at Joint Base San Antonio. Details of the sites and our study methods were published prior to completion of enrollment.^10^ BAMC is the only level I trauma center in the Department of Defense, the largest hospital within the Military Healthcare System, and 1 of 2 level I trauma centers for the southwest region of Texas. UH is a level I trauma center and is the second level I trauma center within the region. Both BAMC and UH are located in San Antonio, Texas, and both are participating members of the Southwest Texas Regional Advisory Council.^11,12^ UCH ED is a level I trauma center in Aurora, Colorado, and a regional receiving center.

### Participants

We prospectively enrolled participants who were known or presumed to be aged 15 years or older at the time of arrival at the enrolling trauma centers who met their highest level of trauma activation (eg, major trauma requiring a full trauma team response) and were injured within 24 hours of arrival. Transfers into our receiving centers were also included if they met the highest activation criteria. Children younger than 15 years and pregnant women were considered exclusions if known at the time of arrival; however, if they were later discovered to be in 1 of these 2 categories, given the observational nature of the study, their data were still used for analysis. If they were in law enforcement custody at the time of arrival or later placed into law enforcement custody during their hospital stay, they were excluded from all analyses.

### Data Collection

Upon launch of the study, trauma laboratory order sets were modified to include iCa values upon arrival and throughout the hospital stay using the laboratory equipment available at each institution. Data were exported from the local electronic medical record systems and uploaded into the Research Electronic Database Capture (REDCap) systems at UH and UCH.^13^ Deidentified data from UH and UCH were then transferred via the API Sync external module to a central REDCap instance at the Vanderbilt University Medical Center (VUMC), which served as the data coordinating center.^14^ BAMC uploaded EHR data directly into the VUMC REDCap instance. Both UH and UCH use the Epic systems. BAMC uses the MHS Genesis custom software system solely for the MHS in partnership with Leidos. Data were reviewed by our study team statistician for missing variables or variables for which a documentation error occurred, which were manually reviewed by a study team member for verification and/or exclusion. Prehospital data were extracted manually by study team members with site-driven quality assurance reviews to ensure accuracy. Additional data were obtained through the local trauma registries. All 3 sites participate in the Trauma Quality Improvement Program and adhere to national trauma registry data standards.^15^ The normal range for iCa for this study was standardized at 4.4 to 5.2 mg/dL (to convert to millimoles per liter, multiply by 0.25) with hypocalcemia and hypercalcemia defined as being outside of that range.

### Statistical Analysis

Deidentified data aggregated from all sites were exported from VUMC REDCap into Excel 365 (Microsoft) and SAS statistical software version 9.4 (SAS Institute) for analysis. Continuous and ordinal variables are presented as medians and IQR and compared using the Wilcoxon rank sum test. Nominal variables are presented as numbers and percentages and compared using the χ^2^ test or Fisher exact test as applicable. Our threshold for statistical significance was *P* < .05 using a 2-sided test or a 95% CI. The Bonferroni correction was applied for multiple comparisons.

## Results

We prospectively enrolled 1270 participants (median [IQR] age, 35 [25-52] years; 999 [79%] male) with an iCa value upon ED arrival. Motor vehicle collisions followed by firearms were the most common injury mechanisms. There were 282 patients (22%) who were hypocalcemic, 925 (73%) who were eucalcemic, and 57 (5%) who were hypercalcemic. Mortality at 24 hours was highest for patients with hypercalcemia (22.8%; 13 patients) followed by those with hypocalcemia (11.9%; 34 patients), and those with eucalcemia (4.3%; 40 patients); the same patterns were seen at 6 hours and discharge (Table 1 and Table 2). The median (IQR) injury severity score was 21 (10-29) for hypocalcemia, 14 (5-25) for eucalcemia, and 22 (13-29) for hypercalcemia. Of those enrolled, 993 were enrolled at BAMC, 183 at UH, and 94 at UCH. Receipt of any blood (binary) product was more frequent among those with calcium derangements; however, there were no significant differences between those with hypocalcemia and those with hypercalcemia (184 patients with hypocalcemia [64.1%], 291 patients with eucalcemia [31.5%], and 38 patients with hypercalcemia [66.7%]) (Table 3). Hypercalcemic patients were more likely than those with hypocalcemia to receive calcium prior to arrival (8 patients [14.0%] vs 4 patients [1.4%) (Table 4). The median iCa upon arrival was generally similar when comparing arrival mode, among patients who did and did not receive blood prior to arrival, and enrollment site (eFigure 1 in Supplement 1). The incidence of hypocalcemia was higher among those who received prehospital blood or fluids, whereas hypercalcemia was higher among those who were transferred (eFigure 2 in Supplement 1). Survival was lower among those with calcium derangements on survival analysis (eFigure 3 in Supplement 1). Mortality at 30 days followed a U-shaped distribution based on arrival calcium levels (Figure).

**Table 1.** Participant Characteristics

Characteristic	Patients, No. (%)			*P* value		
	Hypocalcemic (n = 288)	Eucalcemic (n = 925)	Hypercalcemic (n = 57)	Hypocalcemic vs eucalcemic	Eucalcemic vs hypercalcemic	Hypocalcemic vs hypercalcemic
Sex						
Female	70 (24.3)	189 (20.4)	12 (21.0)	.16	.91	.60
Male	218 (75.7)	736 (79.6)	45 (79.0)			
Age, median (IQR), y	34 (25-51)	35 (25-52)	39 (23-58)	.93	.84	.89
Arrival characteristics						
Emergency medical services	235 (81.6)	773 (83.6)	39 (68.4)	.44	.003^a^	.02
Air	32 (11.1)	92 (1.0)	6 (1.5)	.57	.89	.90
Transfer	24 (8.3)	68 (7.4)	11 (19.3)	.58	.001^a^	.01^a^
Other	2 (0.7)	4 (0.4)	1 (1.8)	.58	.17	.43
Mechanism of injury						
Motor vehicle collision	96 (33.3)	372 (40.2)	29 (5.9)	.04	.11	.01^a^
Gunshot wound	104 (36.1)	221 (23.9)	13 (22.8)	<.001^a^	.85	.05
Other penetrating	33 (11.5)	128 (13.8)	5 (8.8)	.30	.29	.55
Fall	33 (11.5)	116 (12.5)	5 (8.8)	.63	.40	.55
Other blunt	13 (4.5)	47 (5.1)	2 (3.5)	.70	.60	.73
All others	9 (3.1)	41 (4.4)	3 (5.3)	.33	.77	.42
Composite injury severity score, median (IQR)	21 (10-29)	14 (5-25)	22 (13-29)	<.001^a^	<.001^a^	.39
Abbreviated Injury Scale score by body region, median (IQR)						
Head	3 (1-5)	3 (1-4)	2 (1-4)	.40	.09	.31
Neck	0 (0-1)	0 (0-2)	0 (0-2)	.36	.42	.81
Face	1 (0-2)	1 (1-2)	1 (0-1)	.31	.07	.24
Chest	3 (1-3)	3 (1-3)	3 (1-4)	.67	.21	.35
Abdomen	2 (1-3)	2 (1-3)	2 (1-3)	.03	.41	.64
Upper extremity	1 (1-2)	1 (1-2)	2 (1-2)	.12	.49	.92
Lower extremity	2 (1-3)	2 (1-3)	2 (1-3)	.001^a^	.59	.27
Spine	2 (0-2)	2 (0-2)	2 (0-2)	.14	.56	.78
External or skin	1 (0-1)	1 (0-1)	0 (0-1)	.85	.07	.09
Mortality						
At 6 h	24 (8.4)	20 (2.2)	11 (19.3)	<.001^a^	<.001^a^	.01^a^
At 24 h	34 (11.9)	40 (4.3)	13 (22.8)	<.001^a^	<.001^a^	.03
At 30 d	59 (20.6)	119 (12.9)	21 (36.8)	.001^a^	<.001^a^	.008^a^

^a^ *P* < .017 is statistically significant owing to adjustment for multiple comparisons.

**Table 2.** Vital Signs at Emergency Department Arrival

Variable	Median (IQR)			*P* value		
	Hypocalcemic (n = 288)	Eucalcemic (n = 925)	Hypercalcemic (n = 57)	Hypocalcemic vs eucalcemic	Eucalcemic vs hypercalcemic	Hypocalcemic vs hypercalcemic
Heart rate, beats/min	106.0 (88.0-128.0)	100.0 (84.0-118.0)	115.5 (98.0-134.0)	.001^a^	<.001^a^	.03
Systolic blood pressure, mm Hg	110.0 (90.0-130.0)	130.0 (110.0-146.0)	109.0 (9.0-122.0)	<.001^a^	<.001^a^	.68
Diastolic blood pressure, mm Hg	68.0 (58.0-82.0)	79.0 (64.0-90.0)	70.0 (59.0-84.0)	<.001^a^	.008^a^	.94
Temperature, °F	97.6 (96.7-98.2)	97.7 (97.0-98.4)	97.4 (97.0-98.1)	.06	.28	.95
Oxygen saturation, %	97.0 (94.0-99.0)	97.0 (94.0-98.0)	96.0 (90.0-99.0)	.93	.14	.25
Respiratory rate, breaths/min	20.0 (16.0-24.0)	20.0 (17.0-22.0)	22.0 (18.0-24.0)	.10	.006^a^	.08
Glasgow Coma Scale score	14.0 (4.0-15.0)	15.0 (6.0-15.0)	8.0 (3.0-15.0)	.04	.01^a^	.14
Shock index	0.9 (0.7-1.3)	0.8 (0.6-1.0)	1.1 (0.8-1.4)	<.001^a^	<.001^a^	.09

^a^ *P* < .017 is statistically significant owing to adjustment for multiple comparisons.

**Table 3.** 24-Hour Blood Product Consumption

Variable	Patients, No. (%)			*P* value		
	Hypocalcemic (n = 288)	Eucalcemic (n = 925)	Hypercalcemic (n = 57)	Hypocalcemic vs eucalcemic	Eucalcemic vs hypercalcemic	Hypocalcemic vs hypercalcemic
Blood products administered						
Any blood product	184 (63.9)	291 (31.5)	38 (66.7)	<.001^a^	<.001^a^	.76
Whole blood	145 (50.4)	202 (21.8)	31 (54.4)	<.001^a^	<.001^a^	.66
Packed red blood cells	90 (31.4)	134 (14.5)	16 (28.1)	<.001^a^	.006^a^	.75
Plasma	74 (25.7)	117 (12.7)	19 (33.3)	<.001^a^	<.001^a^	.26
Platelets	47 (16.3)	65 (7.0)	10 (17.5)	<.001^a^	.004^a^	.85
Cryoprecipitate	20 (6.9)	27 (2.9)	4 (7.0)	.002^a^	.10	>.99
Total volume, median (IQR), mL						
Whole blood	1500 (900-2070)	1000 (500-2000)	1500 (800-2500)	.08	.10	.47
Packed red blood cells	700 (367-1750)	700 (350-1050)	1750 (800-2275)	.04	.005^a^	.07
Plasma	633 (400-1400)	613 (299-1167)	872 (300-1463)	.29	.68	.89
Platelets	373 (246-600)	282 (223-500)	283 (215-515)	.34	.88	.52
Cryoprecipitate	189 (107-201)	178 (104-200)	425 (130-708)	.71	.17	.17

^a^ *P* < .017 is statistically significant owing to adjustment for multiple comparisons.

**Table 4.** Prehospital Blood, Fluids, and Calcium Administration

Variable	Patients, No. (%)			*P* value		
	Hypocalcemic (n = 292)	Eucalcemic (n = 926)	Hypercalcemic (n = 57)	Hypocalcemic vs eucalcemic	Eucalcemic vs hypercalcemic	Hypocalcemic vs hypercalcemic
Calcium administration	4 (1.4)	15 (1.6)	8 (14.0)	>.99	<.001^a^	<.001^a^
Calcium dose, median (IQR), g	2.0 (2.0-2.5)	1.0 (1.0-2.0)	1.0 (1.0-3.0)	.06	.67	.37
Intravenous fluids administration	81 (28.1)	230 (24.9)	11 (19.3)	.28	.43	.19
Blood products administered						
Any blood product	97 (33.7)	98 (10.6)	19 (33.3)	<.001^a^	<.001^a^	>.99
Whole blood	93 (32.3)	85 (9.2)	19 (33.3)	<.001^a^	<.001^a^	.88
Packed red blood cells	5 (1.7)	14 (1.5)	1 (1.8)	.79	.59	>.99
Plasma	3 (1.0)	7 (0.8)	0	.71	>.99	>.99
Platelets	2 (0.7)	5 (0.5)	0	.67	>.99	>.99
Cryoprecipitate	1 (0.4)	0	0	.24	NA	>.99

Abbreviation: NA, not applicable.

^a^ *P* < .017 is statistically significant owing to adjustment for multiple comparisons.

**Figure.**
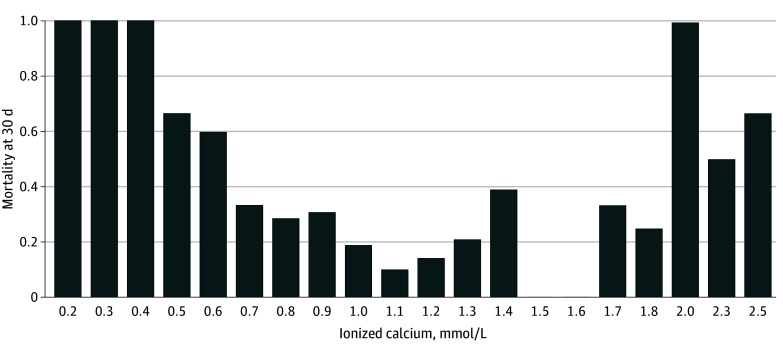
Mortality by Ionized Calcium To convert ionized calcium to milligrams per deciliter, divide by 0.25.

## Discussion

The results of this cohort study are particularly notable for the demonstration of hypercalcemia, which remains poorly described among the studies that assessed calcium derangements upon arrival in trauma patients. On the basis of our overall goal of the study, it is notable that nearly one-fourth of the sample were hypocalcemic on arrival, with nearly one-twentieth hypercalcemic on arrival. The increased mortality at all time points compared with eucalcemia is consistent with previous reports.^5^ However, hypercalcemia was associated with worse mortality than eucalcemia and hypocalcemia. The reason for the findings associated with hypercalcemia remain unclear; however, it may be due to tissue destruction, such as sarcoplasmic reticulum, endoplasmic reticulum, and mitochrondria.^16,17^ This finding is relatively underreported in the trauma literature and represents both a unique and concerning understanding of calcium and trauma physiology. Previous reports on hypercalcemia evaluated the outcomes of prolonged immobilization; given the shorter time from injury to our calcium measurement, this does not explain the findings.^6,18,19^ We did note a higher incidence of motor vehicle collision trauma in the hypercalcemia cohort, suggesting that larger areas of tissue destruction may be a factor.

We must place our findings within the context of the available literature. As noted, a recent 2024 systematic review and meta-analysis analyzed 14 studies.^5^ In comparison to all the studies, we reported on the incidence of hypercalcemia, which is a relatively novel finding. Within that systematic review, 8 of the 14 studies were retrospective, with remaining studies being prospective or a mixture of retrospective and prospective data. The largest prospective study was by Magnotti et al,^20^ which enrolled 591 participants from a single center. Notably, they used a threshold of 1.00 mmol/L, with 16% having hypocalcemia. Although they used a lower threshold than this study, which was based on the laboratory reference range, they also found increased mortality among those with hypocalcemia. They observed a mean injury severity score of 21, which was similar to our dataset. By comparison, our study is a multicenter study, including multiple regions of the country and various climates (eg, Texas vs Colorado). Moreover, as outlined in the Figure, we found a more exaggerated response as the derangements became more profound. Our findings should also be interpreted in the context of derangements of other patients with acute medical conditions, with one study^21^ reporting that 28% of hospitalized patients experienced hypocalcemia and 5% experienced hypercalemia, suggesting that derangements may occur as a result of acute stress in general.

The literature on hypercalcemia after traumatic injury is sparse, mostly related to excess administration of calcium during resuscitation. Circulating hypotheses regarding the association of hypercalcemia and adverse outcomes after trauma stem from retrospective studies of European trauma registries and a retrospective study by MacKay et al^6^ in Philadelphia. The European studies demonstrated that hypercalcemia upon arrival to the ED was associated with the highest mortality rates when compared with eucalcemic or hypocalemic patients.^22^ Hypercalcemic patients in the study by Mackay et al^6^ also exhibited drastically higher mortality, where hypercalcemia was defined as iCa greater than 1.30 mmol/L.^22^ After injury or other periods of physiologic stress, acidosis stimulates parathyroid hormone production, leading to elevated calcium levels. Relevant to parathyroid dysfunction, a previous study found an association between parathyroid dysfunction and mortality, suggesting that future studies should investigate this further.^23^ For this among other reasons, such as the vital role that calcium plays in coagulation and platelet function, it has been proposed that hypocalcemia be added to the current lethal triad of acidosis, hypothermia, and coagulopathy.^2^ However, given the lethal triad is contingent on iatrogenic causes, these findings suggest calcium derangements are multifactorial and therefore may not be indicated to add to the lethal triad. To that end, with the potential signal for harm from empiric calcium, the administration of calcium should, according to our data, be relegated to address citrate toxicity and/or be driven by point-of-care testing as it becomes more widely available.

### Limitations

Our study has several limitations, including the observational design as noted above regarding the association between calcium derangements and outcomes. First, the majority of patients were enrolled at BAMC, which may have potentially skewed the results and limited generalizability. However, our sample does include multiple centers, which would enhance the generalizability. Second, we did not capture data on patients who did not have an iCa drawn upon arrival to the ED, which can occur for a variety of reasons, including delays in upgrading their trauma status to the highest level per institutional policy, point-of-care testing unavailability, which is particularly notable at BAMC, since all of our iCa measurements are performed with point-of-care machines, or other clinically indicated reasons for deviating from the study implemented order set. As such, we cannot describe the patients who were not captured. Third, we rely on information being appropriately documented, particularly with the prehospital data, which were derived from review of outside hospital medical records if they were transferred and/or records from emergency medical services personnel, some of which is handwritten.

However, despite the size of our study, we must note that this was observational in design, which limits our ability to draw definitive conclusions regarding empiric administration of calcium. To that end, we must discuss the on-going Calcium and Vasopressin Following Early Resuscitation trial that is funded by the Department of Defense and will use empiric calcium as part of the resuscitation strategy for patients undergoing blood product resuscitation. This study is ongoing with an expected completion in 2028 and an estimated enrollment size of 1050. This interventional trial will shed further light on calcium derangements and the potential benefits or deleterious effects of empiric calcium administration. Additional preplanned analyses will help illuminate possible physiological mechanisms.

## Conclusions

Hypercalcemia was less common than hypocalcemia; however, it was associated with worse mortality at all time points. Blood product consumption was higher among those with hypercalcemia or hypocalcemia compared with eucalcemia but were similar among those with any calcium derangement. Prospective interventional trials are needed to understand the implications of empiric treatment.

## Notes

Supplement 1. eFigure 1. Median ionized calcium on arrival eFigure 2. Incidence of calcium derangement on arrival eFigure 3. Survival plots

Supplement 2. Data Sharing Statement
