# Error in Table

**DOI:** 10.1001/jamanetworkopen.2026.11311

**Published:** 2026-04-15

In the Original Investigation titled “Initial Calcium Derangements in Major Trauma and Outcomes,”^1^ published February 25, 2026, there was an error in Table 1. In the eucalcemic column, the percentage of patients with a motor vehicle collision should have been 40.2%, not 4.2%. This article has been corrected.^1^
