# Supplementary material for: Initial Calcium Derangements in Major Trauma and Outcomes
Source: JAMA Netw Open. 2026 Feb 25;9(2):e260083. doi: 10.1001/jamanetworkopen.2026.0083 (PMC12936877; doi:10.1001/jamanetworkopen.2026.0083)
Supplement: Supplement 1. — eFigure 1. Median ionized calcium on arrival eFigure 2. Incidence of calcium derangement on arrival eFigure 3. Survival plots [file jamanetwopen-e260083-s001.pdf]

Supplemental Online Content

Schauer SG, Nicholson SE, Rizzo JA, et al. Initial calcium derangements in major trauma and outcomes. *JAMA Netw Open*. 2026;9(2):e260083. doi:10.1001/jamanetworkopen.2026.0083

eFigure 1. Median ionized calcium on arrival
eFigure 2. Incidence of calcium derangement on arrival
eFigure 3. Survival plots

This supplemental material has been provided by the authors to give readers additional information about their work.

© 2026 Schauer SG et al. *JAMA Network Open*.

eFigure 1. Median ionized calcium on arrival

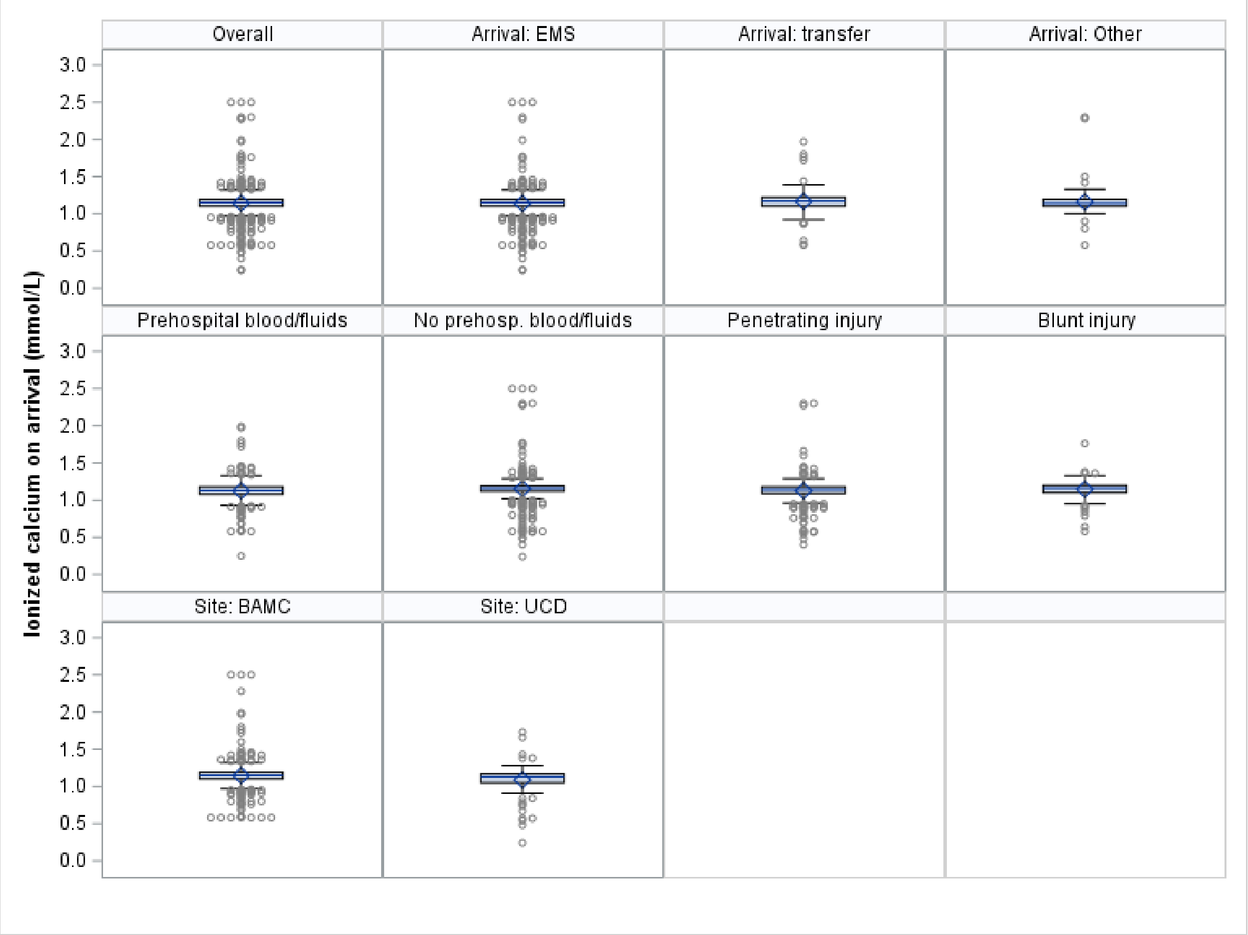

© 2026 Schauer SG et al. *JAMA Network Open*.

eFigure 2. Incidence of calcium derangement on arrival

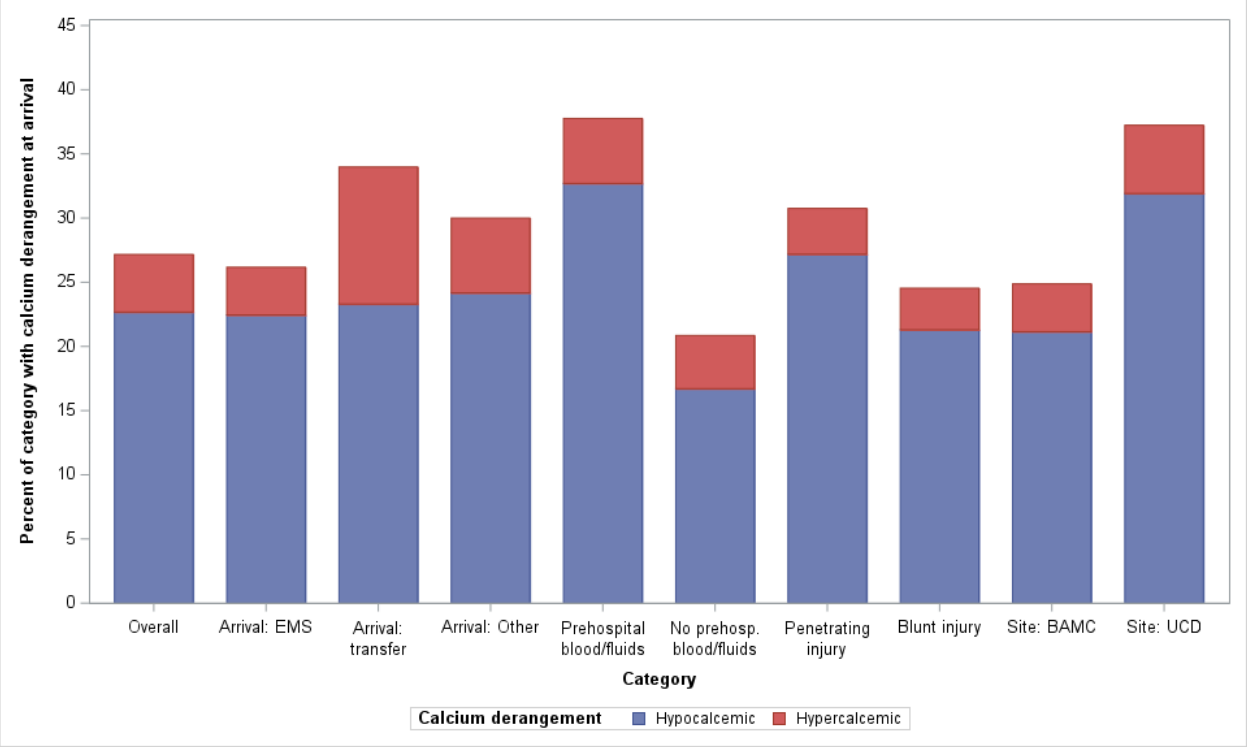

© 2026 Schauer SG et al. *JAMA Network Open*.

eFigure 3. Survival plots

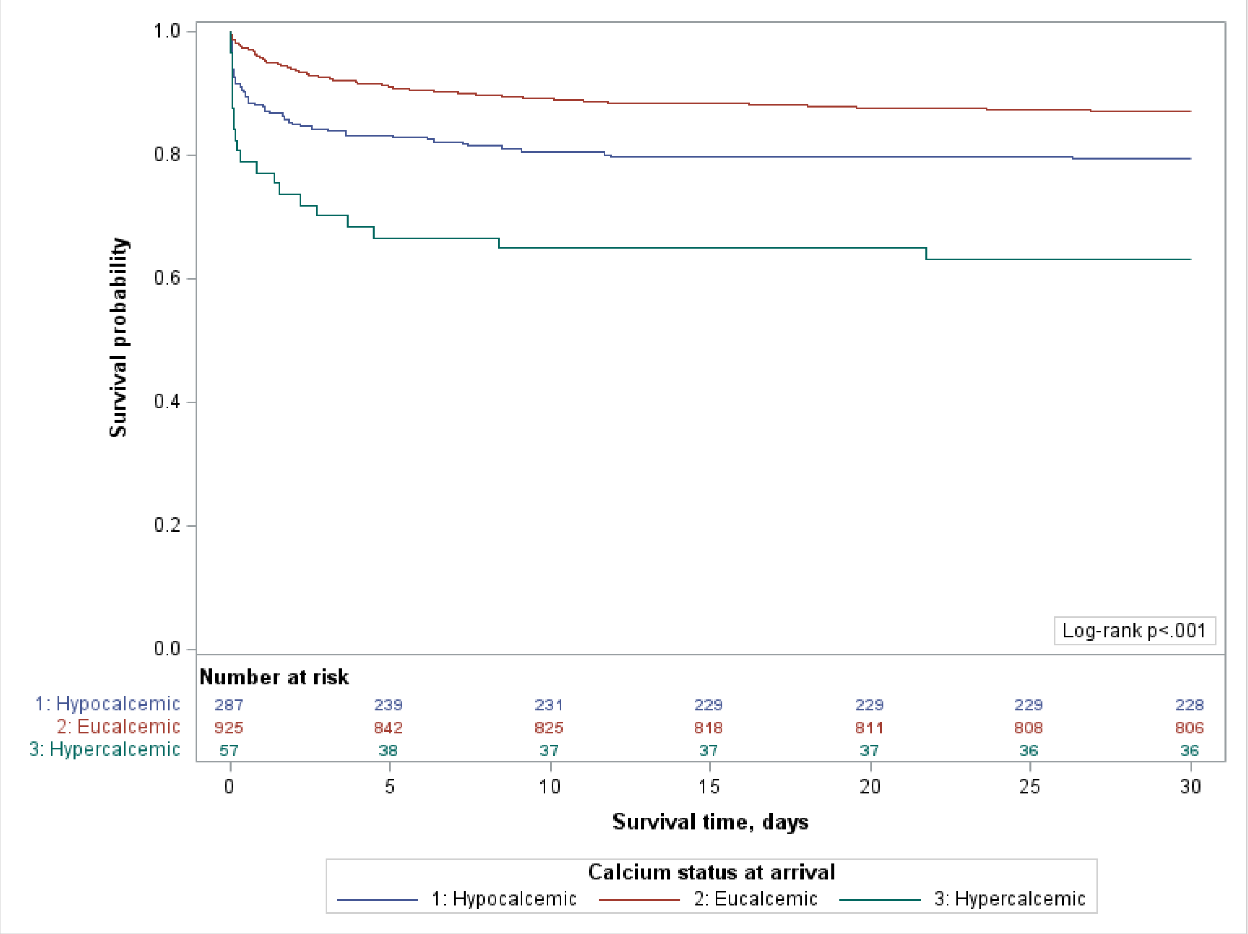

© 2026 Schauer SG et al. *JAMA Network Open*.

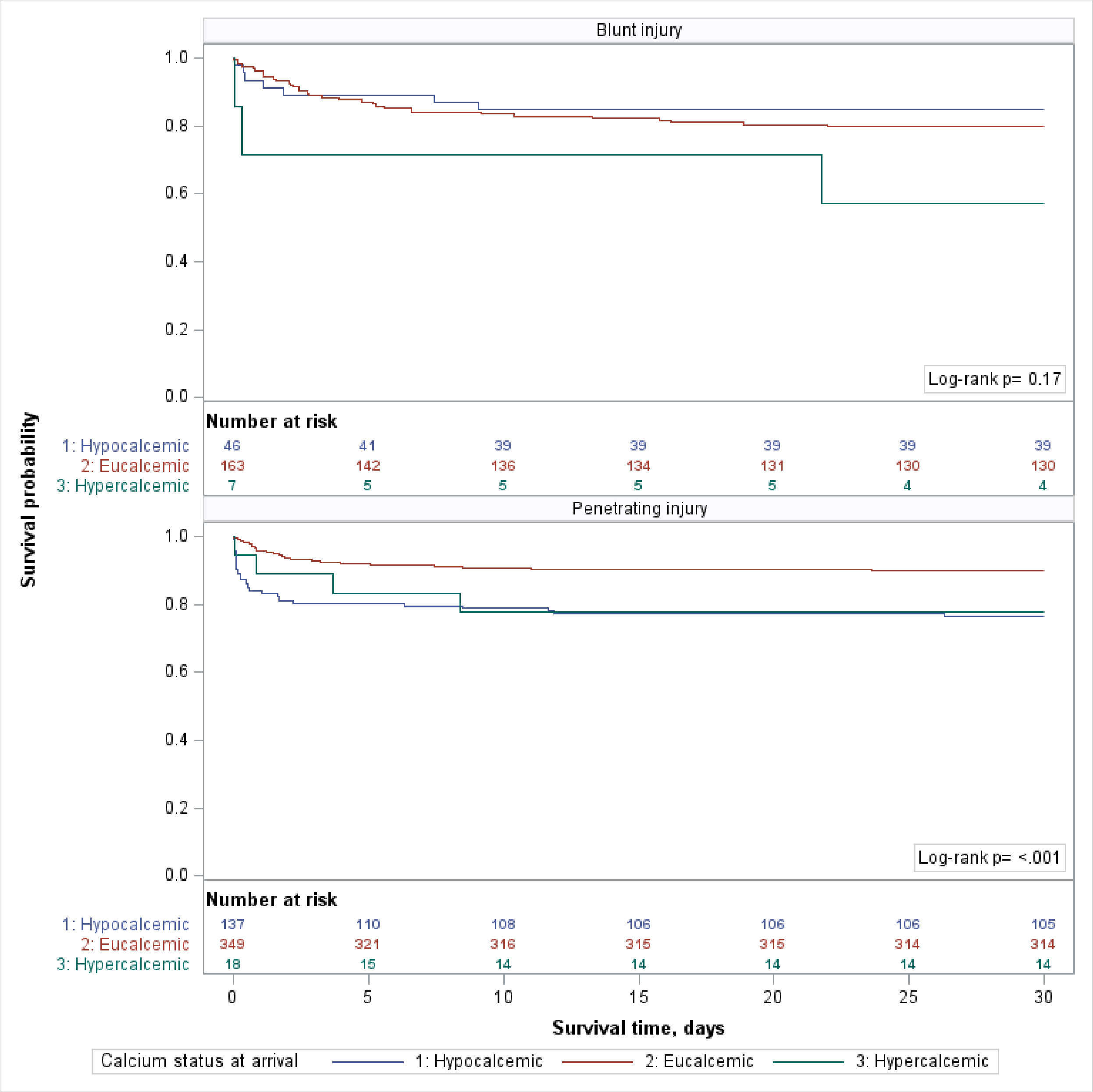

© 2026 Schauer SG et al. *JAMA Network Open*.
