# Supplementary material for: Initial Calcium Derangements in Major Trauma and Outcomes
Source: JAMA Netw Open. 2026 Feb 25;9(2):e260083. doi: 10.1001/jamanetworkopen.2026.0083 (PMC12936877; doi:10.1001/jamanetworkopen.2026.0083)
Supplement: Supplement 2. — Data Sharing Statement [file jamanetwopen-e260083-s002.pdf]

Data Sharing Statement

Schauer. Initial Calcium Derangements in Major Trauma and Outcomes. *JAMA Netw Open*. Published February 25, 2026. doi:10.1001/jamanetworkopen.2026.0083

Data
Data available: No

Additional Information
Explanation for why data not available: The Defense Health Agency requires appropriate data sharing agreements before the data can be shared externally.
